# How, why, and under what circumstances can supportive supervision programs improve malaria case management? A realist program theory

**DOI:** 10.1093/heapol/czaf020

**Published:** 2025-03-26

**Authors:** Fatuma Manzi, Jessie K Hamon, Mena K Agbodjavou, Jenna Hoyt, August Kuwawenaruwa, Yusufu Kionga, Christian Agossou, Abdunoor M Kabanywanyi, Christelle Boyi-Hounsou, Abdallah Lusasi, Samwel Lazaro, Ramani Saliou, Augustin Kpemasse, Erik Reaves, Chonge Kitojo, Ahmed Saadani Hassani, Virgile Gnanguenon, Jean-Paul Dossou, Jayne Webster

**Affiliations:** U.S. President’s Malaria Initiative (PMI), PMI Insights Consortium, 437 N 34th Street, Seattle, WA 98103, United States; Ifakara Health Institute, Kiko Ave, Plot 463, Mikocheni, Dar es Salaam, Tanzania; U.S. President’s Malaria Initiative (PMI), PMI Insights Consortium, 437 N 34th Street, Seattle, WA 98103, United States; London School of Hygiene & Tropical Medicine, Keppel Street, London WC1E 7HT, United Kingdom; U.S. President’s Malaria Initiative (PMI), PMI Insights Consortium, 437 N 34th Street, Seattle, WA 98103, United States; Centre de Recherche en Reproduction Humaine et en Démographie, Rue 903, Carré 129 Patte d’Oie, Cotonou, Bénin; U.S. President’s Malaria Initiative (PMI), PMI Insights Consortium, 437 N 34th Street, Seattle, WA 98103, United States; London School of Hygiene & Tropical Medicine, Keppel Street, London WC1E 7HT, United Kingdom; U.S. President’s Malaria Initiative (PMI), PMI Insights Consortium, 437 N 34th Street, Seattle, WA 98103, United States; Ifakara Health Institute, Kiko Ave, Plot 463, Mikocheni, Dar es Salaam, Tanzania; U.S. President’s Malaria Initiative (PMI), PMI Insights Consortium, 437 N 34th Street, Seattle, WA 98103, United States; Ifakara Health Institute, Kiko Ave, Plot 463, Mikocheni, Dar es Salaam, Tanzania; U.S. President’s Malaria Initiative (PMI), PMI Insights Consortium, 437 N 34th Street, Seattle, WA 98103, United States; Centre de Recherche en Reproduction Humaine et en Démographie, Rue 903, Carré 129 Patte d’Oie, Cotonou, Bénin; U.S. President’s Malaria Initiative (PMI), PMI Insights Consortium, 437 N 34th Street, Seattle, WA 98103, United States; Centre de Recherche en Reproduction Humaine et en Démographie, Rue 903, Carré 129 Patte d’Oie, Cotonou, Bénin; U.S. President’s Malaria Initiative (PMI), PMI Insights Consortium, 437 N 34th Street, Seattle, WA 98103, United States; Centre de Recherche en Reproduction Humaine et en Démographie, Rue 903, Carré 129 Patte d’Oie, Cotonou, Bénin; Ministry of Health, Community Development, Gender, Elderly & Children, National Malaria Control Program, PO Box 743, Dodoma, Tanzania; Ministry of Health, Community Development, Gender, Elderly & Children, National Malaria Control Program, PO Box 743, Dodoma, Tanzania; Ministère de la Santé, Programme National de Lutte Contre le Paludisme, Rue 1684, Sodjatinmé, Cotonou, Bénin; Ministère de la Santé, Programme National de Lutte Contre le Paludisme, Rue 1684, Sodjatinmé, Cotonou, Bénin; U.S. PMI, Centers for Disease Control and Prevention, 686 Old Bagamoyo Road, Dar es Salaam, Tanzania; U.S. PMI, U.S. Agency for International Development, 686 Old Bagamoyo Road, Dar es Salaam, Tanzania; U.S. PMI, Centers for Disease Control and Prevention, Marina Avenue, Cotonou, Benin; U.S. PMI, U.S. Agency for International Development, Marina Avenue, Cotonou, Benin; U.S. President’s Malaria Initiative (PMI), PMI Insights Consortium, 437 N 34th Street, Seattle, WA 98103, United States; Centre de Recherche en Reproduction Humaine et en Démographie, Rue 903, Carré 129 Patte d’Oie, Cotonou, Bénin; U.S. President’s Malaria Initiative (PMI), PMI Insights Consortium, 437 N 34th Street, Seattle, WA 98103, United States; London School of Hygiene & Tropical Medicine, Keppel Street, London WC1E 7HT, United Kingdom

**Keywords:** supportive supervision, malaria, case management, realist evaluation, program theory, Tanzania, Benin

## Abstract

Supportive supervision (SS) programs aim to enhance the quality of care by strengthening the performance of health providers. Commonly part of broader quality improvement efforts, SS programs are increasingly used in low-and middle-income countries to improve malaria case management. Despite substantial investments and some positive outcomes, little is known about what drives their effectiveness. A realist evaluation was conducted in Tanzania and Benin to explain how, why, and under what circumstances SS programs can improve the facility-based management of uncomplicated malaria in children <5 years. A program theory was developed through a team-based analysis of empirical data collected in both countries at two time points. Data included 218 in-depth and 12 structured interviews with stakeholders, 154 audits of febrile case management decisions, and 4 health facility audits. Stakeholder perspectives identified three acceptability mechanisms driving SS program outcomes in the studied contexts: the affective attitude, self-efficacy, and burden of the program as perceived by key actors. The pathway through which these mechanisms were perceived to shape malaria case management (diagnosis and treatment) practices was defined by the (i) extent to which the program was integrated into the public health system; (ii) frequency with which SS visits were conducted by appropriate supervisors; (iii) degree to which supervisors coached, rather than policed, supervisees; and (iv) level of collaboration achieved between supervisees and supervisors. The program actors’ perception of the program’s effectiveness was also found to be crucial to its sustainability. This study explains the dynamics driving SS program outcomes and underscores the role played by the cognitive and emotional responses of program actors. These insights are likely to be transferable to other settings with similar contexts and can help inform the design, implementation, monitoring, and evaluation of new and ongoing SS programs

Key messages

- According to experienced stakeholders, supportive supervision programs can improve the diagnosis and treatment of malaria in children <5 years of age, provided the programs are well integrated into the public health system and health providers receive regular supervision from qualified professionals who adopt a collaborative approach.
- Supportive supervision program outcomes are driven by the attitudes and emotional responses of the actors involved. In certain contexts, factors such as their confidence in their abilities, their perceived workload, and their belief in the program’s effectiveness play a critical role in shaping outcomes.
- Decision-makers seeking to strengthen supportive supervision programs should focus on understanding how the program’s contexts, components, and actors’ responses interact. This understanding can help them identify leverage points within the program where small adjustments can enhance actors’ responses, and in turn lead to better outcomes.

## Background

According to the World Malaria Report, 74.5% of the global malaria deaths in 2022 occurred among children <5 years of age in the World Health Organization’s (WHO) African Region, in part due to persisting gaps in the quality of malaria case management (World Health Organization 2023). Household surveys performed across 22 countries in the region between 2015 and 2022 revealed that only 54% of children <5 years who sought care for fever received a diagnosis through a finger or heel prick, and 65% of children who were treated with an antimalarial received artemisinin-based combination therapy (ACT)—the WHO recommended treatment for uncomplicated malaria in children (World Health Organization 2023). Thus, enhancing and maintaining improvements to the quality of malaria case management remain public health priorities in many countries. For instance, the latest demographic and health survey (DHS) in Benin (2017–18) revealed that whilst 97.5% of febrile children <5 years of age who sought care in the 2 weeks preceding the survey were tested for malaria using a rapid diagnostic test, only 37.0% of children received an ACT among those who were given an antimalarial (The DHS Program 2020). In contrast, the corresponding figures in Tanzania (2022) were 96.9% and 94.7%, respectively [Ministry of Health (MoH) (Tanzania Mainland) Ministry of Health (MoH) (Zanzibar) National Bureau of Statistics (NBS) Office of the Chief Government Statistician (OCGS) and ICF, 2022], reflecting recent improvements.

Building on insights from broader quality improvement initiatives, supportive supervision (SS) programs have gained traction over the past two decades as a strategy to improve malaria case management in sub-Saharan Africa. These programs aim to bolster the quality of health services by strengthening the knowledge and skills of health providers (Bailey et al. 2016). While the specific implementation of SS programs varies across settings, they generally focus on fostering two-way communication between health providers and experienced professionals in supervisory roles (Garrison et al. 2004, Kilminster et al. 2007, Avortri et al. 2019, Barat et al. 2023). Often targeting underperforming health facilities (Ssekabira et al. 2008, Odhiambo et al. 2017, Worges et al. 2018, Eliades et al. 2019a, Gidey et al. 2021), these programs use readiness and competency-based checklists to assess clinical and/or laboratory practices. Following assessments, supervisors typically provide on-site coaching to address identified gaps (Namagembe et al. 2012, Bello et al. 2013, Burnett et al. 2016, 2019, Luckett et al. 2016, Worges et al. 2018, Martin et al. 2019, Eliades et al. 2019a, 2019b, Gidey et al. 2021).

Research indicates that the implementation of SS programs is influenced by several factors, including the frequency of visits, the selection and quality of supervisory tools, and the availability of qualified supervisors (Bello et al. 2013, Burnett et al. 2016, Eliades et al. 2019a, Khan et al. 2011, Luckett et al. 2016, Namagembe et al. 2012; Rowe et al. 2003, Worges et al. 2018). While some evidence suggests that SS [and outreach training and supportive supervision (OTSS)] can enhance malaria diagnosis and treatment practices (Alombah et al., 2019, Bello et al. 2013, Worges et al. 2018, Eliades et al. 2019b, Ashton et al. 2023, Tropical Health 2023), little is known about what drives the effectiveness of these programs (PMI Insights SS PE Team 2022). This gap in knowledge mirrors the broader literature on SS aimed at improving a range of primary health services in low- and middle-income countries beyond malaria case management, where evidence on the impact of SS on clinical competence and outcomes is mixed (Akila et al. 2024, Avortri et al. 2019, Bailey et al. 2016, Deussom et al. 2022, Leslie et al. 2016; Rowe et al. 2010; 2022).

Furthermore, there is a paucity of theories or frameworks that explain the mechanisms and pathways through which SS programs work to improve primary health services in general, and malaria case management more specifically. This knowledge gap is partly due to the complex, targeted and reactive nature of SS programs, which increases the challenges of evaluating both their effectiveness and the factors driving their effectiveness. With the aim of targeting underperforming health facilities, the focus, content, and timing of SS activities are fluid, making more static evaluations inherently difficult, with findings from cross-sectional studies under-estimating the effect of SS due to dilution from nontargeted health facilities. To address this knowledge gap, a realist evaluation was carried out between 2021 and 2023 to develop an evidence-based program theory that outlines how, why, and under what circumstances SS programs can improve the facility-based management of uncomplicated malaria in children <5 years of age.

## Methods

### Study design

The study consisted of realist evaluations of SS programs in Tanzania and Benin. Realist evaluation is designed to uncover the pathways that drive intervention outcomes. It is founded on the principle that an intervention, such as SS, generates outcomes via mechanisms (Marchal et al. 2010). Here, mechanisms refer to the cognitive or affective responses of actors to the intervention. These mechanisms are usually nonobservable attributes and are influenced by the contexts in which they are activated (Astbury and Leeuw 2010). The realist evaluations conducted in both countries sought to identify the mechanisms and conditions that drive SS program outcomes.

Two rounds of data collection were conducted in Tanzania and Benin to explore the perspectives of key stakeholders regarding the design and implementation of SS programs. These perspectives, founded on practical experiences, shed light on the contexts, mechanisms, and outcomes of the SS programs, which enabled the development and refinement of a program theory.

### Description of SS programs studied

In Tanzania, health providers working in health facilities received SS from trained Council Health Management Team (CHMT) members, who, in turn, were overseen by Regional Health Management Team (RHMT) members and the National Malaria Control Program (NMCP). SS visits were designed to be carried out in health facilities on a quarterly basis by CHMT supervisors.

In Benin, two SS programs were implemented to improve malaria case management: one led by the NMCP that focused on enhancing the quality of laboratory practices; and the other supported by a nongovernmental organization (NGO) that was dedicated to improving the quality of clinical practices. These programs were designed for health providers (clinician and laboratory technicians) to receive SS visits from health zone personnel. In turn, supervisors from the health zones received guidance from departmental- and national-level actors. All laboratories and health facilities were expected to receive SS visits semi-annually and annually, respectively (25% of all facilities were targeted each quarter). In addition, during each quarter, 5% of the lowest performing health facilities were also expected to be visited.

In both countries, supervisors were expected to assess health facilities, laboratories, and health providers using standardized checklists to generate performance scores. Underperforming health facilities were meant to be prioritized for SS visits based on these scores and other routinely collected data. Supervisors were also expected to observe health providers’ practices during consultations, laboratory investigations, and drug dispensation to provide feedback on appropriate practices. To guide this process, paper-based checklists were used in Benin, whilst an electronic data capture system, the malaria service and data quality improvement (MSDQI) package, was used in Tanzania (Hussein et al. 2023). At the end of each SS visit, supervisors were expected to develop written reports, problem-solving or quality improvement plans. This report or plan was intended to summarize the performance shortcomings, and the operational bottlenecks uncovered during the visit and outline the corrective actions needed to remedy these.

### Study sites

The study was conducted in 4 of 12 departments in Benin and 5 of 184 councils in Tanzania. To safeguard participant confidentiality, detailed study site information cannot be disclosed. The departments and councils included in the study were located in both rural and peri-urban environments where SS programs had been active for over 2 years and encompassed a range of geographical and population sizes. In each council and department, two health facilities (specifically, health centers) were purposively selected to capture data from facilities with a mix of high and low attendance by children <5 years of age, febrile cases tested for malaria, and previous SS visit performance scores. During data collection, two of the eight health facilities in Benin were found to have nonfunctioning laboratories. In total, the study included 18 health facilities where the NGO-supported SS program was implemented and 16 laboratories where the NMCP-led SS program was implemented.

**Figure 1.**
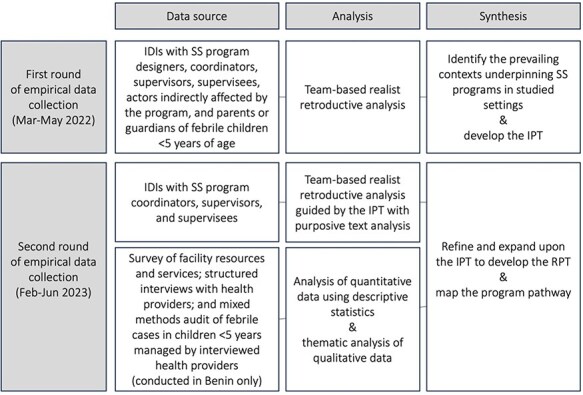
Summary of methods.

As indicated in Fig. 1, empirical data collection was conducted from March to May 2022 and February to June 2023 in each country. In each round of data collection, in-depth interviews (IDIs) with key stakeholders were carried out. Guided by SS program designers and implementers, maximum variation purposive sampling was used to select a range of stakeholders for these interviews. Eligible stakeholders were individuals ≥18 years who designed, delivered, received, or were affected by, the SS programs Although most interviews were carried out in selected health facilities, interviews with national and sub-national actors were held in administrative offices.

### Data collection

In the first round of data collection, IDIs were conducted in both countries with: SS program designers, coordinators, supervisors, supervisees, actors indirectly affected by the program (e.g. health facility managers), and parents or guardians seeking care for febrile children <5 years of age. These participants were asked a series of open-ended questions focused on the program’s contexts, components, perceived outcomes, and the factors believed to influence the program’s effectiveness, with the aim of developing an initial program theory (IPT).

In the second round, IDIs were conducted with SS program coordinators, supervisors, and supervisees. Using thematic, example-driven, and theory-testing questions, these IDIs interrogated elements of the IPT and related themes, including: (i) the programs’ facility targeting strategies; (ii) the training and supervision of supervisors; (iii) the supervisor–supervisee interpersonal dynamics; (iv) the guidance exchanged during SS visits; (v) the perceived effect of the programs on malaria diagnosis and treatment outcomes; and (vi) the potential mechanisms driving the effectiveness of SS programs in both countries. In Benin, where health providers were better able to distinguish the SS program from other types of supervision, the IDIs were supplemented by additional data collection to enrich the program theory. This included an audit of facility resources and services; structured interviews with health providers who received SS in the previous year; and an audit of febrile cases in children <5 years managed by these same providers. The case audit included closed questions on the decisions made by health providers in the diagnosis and treatment of each case, along with four open-ended questions that explored the factors influencing these decisions. A health facility was purposively selected for this audit from each of the four departments studied in Benin with the intent to capture some diversity in facility size and proximity to urban areas.

### Data management and analysis

Audio recordings of IDIs were transcribed *verbatim* by members of the research team. Transcripts from interviews performed in Tanzania were translated from Swahili to English to facilitate the analysis, while transcripts from interviews conducted in Benin were analyzed in their original language (French). These transcripts were imported into Nvivo 1.7.1 for coding and analysis. Data from the health facility audit, structured interviews with health providers, and case audits conducted in Benin were imported into Excel for cleaning and analysis. All data were anonymized and labelled with the type of respondent prior to being analyzed. Once the analyses were complete, quotes were extracted from the data for illustrative purposes.

A team-based approach to realist retroductive analysis was used to analyze the data from the first round of IDIs. Briefly, this approach involved researchers from each of the three research institutions represented within the core research team identifying key contexts, mechanisms, and outcomes from the data. This was achieved by (i) examining the interviewers’ field notes, (ii) conducting a holistic reading of transcripts, and (iii) directly coding all transcripts. Following each of these three steps, the researchers held several discussions, facilitated by the creation of mind maps, to gradually shape the IPT. The IPT, organized into context–intervention–actor–mechanism–outcome (CIAMO) configurations, offered a preliminary explanation of the effect of SS programs for malaria case management (diagnosis and treatment) practices (program outcomes), the causes of this effect (mechanisms), and the circumstances that shaped these effects (contexts).

The analysis of the second round of IDIs was guided by the IPT and was initiated through twice-weekly discussions (whilst data were collected) involving the data collection and research team members. This enabled the researchers to become familiar with the data collectors’ interpretation of the data. Subsequently, IDI transcripts were examined holistically to identify prominent themes within the data, and deductive coding was performed. The coding focused on the SS programs’ contexts, components, actors, mechanisms, and reported outcomes. Particular attention was given to data that supported, expanded, or refuted the IPT. Where the interpretation of the data proved challenging, a consensus among the researchers was sought through discussion. This involved deliberating on the most plausible explanations for observed patterns, drawing on the collective expertise of the team and considering alternative explanations, as well as the relative weight of different data.

Additionally, quantitative data from the health facility audit and the structured interviews with health providers carried out in Benin were summarized using descriptive statistics. This involved summarizing health facility resources and services, and the health providers’ characteristics and experiences. We defined malaria case management in line with national guidelines on diagnosis and treatment. Case audit data of febrile children were organized into an effectiveness cascade, which included the diagnosis and treatment of malaria test-positive and -negative cases (Webster et al. 2014). This cascade was complemented by a thematic analysis in Excel of open-ended audit questions, which explained the decisions made by individual health providers in the management of each case (e.g. their reasons for adhering or deviating from malaria diagnosis and treatment guidelines and the influence that past SS visits had on these decisions). Results from these analyses were cross-checked with findings from the IDIs to identify gaps or contradictions, in an effort to strengthen the robustness of the program theory.

Together, the findings from these analyses (summarized in Supplementary Materials) were synthesized into a revised program theory (RPT) (Kim and Andersen 2012), which explained how, why, and under what circumstances SS programs can improve the facility-based management of malaria in children <5 years of age. Furthermore, purposive text analysis of interview transcripts was carried out to map the causal relationships between the elements of the RPT (i.e. the program pathway), providing further insights into the driving forces behind program outcomes and the underlying dynamics of SS programs (Kim and Andersen 2012, Geng et al. 2022). Additionally, the outputs from this work, including the RPT, were reviewed and confirmed by interested parties in both countries through collaborative workshops, which included program designers, implementers and key decision-makers in the public health sector.

Here, we present the prevailing contexts anchoring the program theory, and we describe the pathways and mechanisms that drive the outcomes of SS programs that target the improvement of malaria case management practices in the studied contexts.

## Results

### Respondent characteristics

In total, 218 IDIs were conducted across both countries, with 144 and 74 IDIs performed during the first and second rounds of data collection, respectively (Table 1). The majority of SS program designers who took part in the IDIs operated at the national level, while coordinators and supervisors were primarily situated at regional and council (Tanzania), and departmental (Benin) levels. Supervisees included both health providers who were tasked with either the clinical management of febrile children or who routinely conducted malaria rapid diagnostic tests (mRDTs) or malaria microscopy in health facilities. Additionally, in Benin, four health facility audits were carried out, 12 health providers (supervisees) participated in structured interviews, and 154 cases of febrile children <5 years managed by these same providers were audited.

**Table 1.** Summary of in-depth interviews respondents

Respondent	Description of respondents	IDIs in Tanzania		IDIs in Benin		Total (*N* = 218)
		Round 1 (*n* = 80)	Round 2 (*n* = 32)	Round 1 (*n* = 64)	Round 2 (*n* = 42)	
Designer	Actor who influenced the design of the SS programs	5	-	3	-	8
Coordinator	Actor who oversaw the supervisors’ activities (supervisor of supervisor)	2	2	4	6	14
Supervisor	Actor who supervised health providers tasked with malaria case management in health facilities	10	13	10	14	47
Supervisee	Health provider tasked with malaria case management in children under five years and who received SS	25	17	15	22	79
Client	Parent or guardian of febrile children under five years who sought care in health facilities	18	-	8	-	26
Other actor	Actor indirectly affected by SS activities in health facilities (e.g. health facility in-charge)	20	-	24	-	44

### Prevailing contexts

Several contexts were found to shape the design and implementation of SS programs in Tanzania and Benin. Macro-level contexts were that (i) improving the management of malaria in children <5 years of age was a national priority and championed by the country’s Ministry of Health (MoH); (ii) financial and technical support to the MoH for the SS programs were largely provided by an external partner; and (iii) mid-level managers within the health system who acted as SS coordinators and supervisors had heavy workloads and limited transportation to carry out SS visits. For instance, a program designer in Benin highlighted that:

to put the funding in place to be able to carry out [SS] activities … you have to go through American NGOs … and often these NGOs come together in a consortium … Well, now by forming a consortium, there is always someone who is the lead, who always has the big part. So, they are the ones who now have the governance of the activities (Designer 01, Round 1, Benin).

Meso-level contexts were that (i) health facilities were often understaffed and at times lacked malaria diagnosis and treatment supplies, and (ii) not all health providers responsible for malaria case management in health facilities had the necessary training to fulfill their role. A coordinator in Tanzania explained this context by stating that in health facilities:

for some [health providers], adherence to the guidelines becomes a problem because of not having skills…[and] you find that [they] are overwhelmed so they cannot comply well with the guidelines, [and] another thing is that there could be shortage maybe of commodities or stock out of commodities, but it is very rare (Coordinator 02, Round 2, Tanzania).

The prevailing micro-level context was that parents and guardians commonly delayed seeking care for febrile children or attempted to influence the service provision process due to low purchasing power, cultural beliefs, or misinformation about malaria. For example, a supervisee in Benin explained that:

if [parents] can’t pay for the products, [they] push against it if we ask for tests. They say there is no money. We can’t do the tests [and they ask us to] prescribe medication and they’ll at least manage to pay that (Supervisee 17, Round 1, Benin).

### Initial program theory

Based on the findings from the first round of empirical data collection in Tanzania and Benin, the IPT encompassed elements of both the design and implementation of SS programs and consisted of three CIAMO configurations (available in Supplementary Materials). Overall, the IPT postulated that the SS programs’ main actors’ perceptions of the program were likely to be central to the outcomes of SS programs in the studied contexts.

### Revised program theory

The RPT consisted of four CIAMO configurations (Box 1), which confirmed and expanded upon the central idea postulated by the IPT. Specifically, the RPT demonstrated that the outcomes of SS programs in the studied contexts were driven by three key mechanisms: the affective attitude, self-efficacy, and burden of the program as perceived by its main actors.

Box 1. **Revised program theory**

1. In a context where improving malaria case management is a national priority and where financial and technical support to the Ministry of Health for the SS program is provided by external partners **(C)**, if the program is integrated into the public health system **(I)**, then SS coordinators and supervisors **(A)** will perceive the program to be credible, relevant, and important (**M: affective attitude**) and will accept the workload associated with their SS roles (**M: perceived burden**), so they will carry out SS activities regularly **(O)**.

1. In a context where SS supervisors are mid-level managers and have heavy workloads **(C)**, if they receive the tools and training to execute their role and regularly conduct SS visits **(I)**, then these supervisors **(A)** will be confident in their ability to perform their role **(M: self-efficacy)**, so they will create a supportive learning environment for supervisees **(O)**.
2. In a context where not all health providers have the training to fulfil their role and most face resource or client-related constraints, which hinder their adherence to guidelines **(C)**, if supervisors coach, rather than police, supervisees **(I)**, then these supervisees **(A)** will not feel pressured during SS visits **(M: perceived burden)** and will value these visits as learning opportunities **(M: affective attitude)**, so they will be willing to collaborate with supervisors **(O)**.

1. In a context where health facilities are regularly understaffed and at times experience shortages of malaria diagnosis and treatment supplies **(C)**, if supervisors collaborate with supervisees to develop and implemented quality improvement plans **(I)**, then supervisees **(A)** will be confident in their ability to implement their supervisors’ guidance **(M: self-efficacy)**, so they will make changes to their malaria diagnosis and treatment practices **(O)**.

C—context; I–intervention; A—actor; M—mechanism; O—outcome

### Program pathway mapping

**Figure 2.**
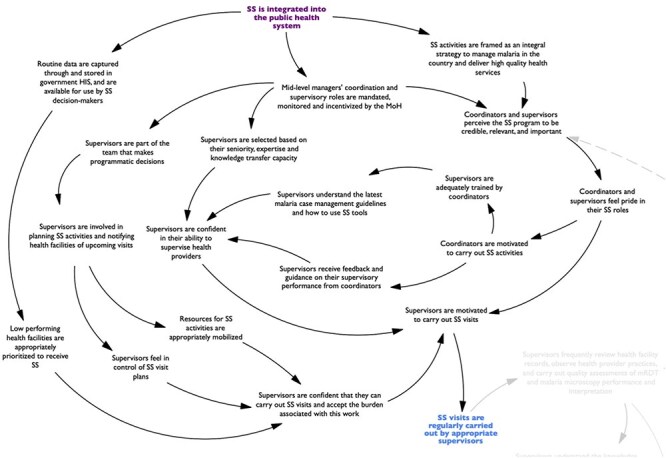
Map of the program pathway (first section).

**Figure 3.**
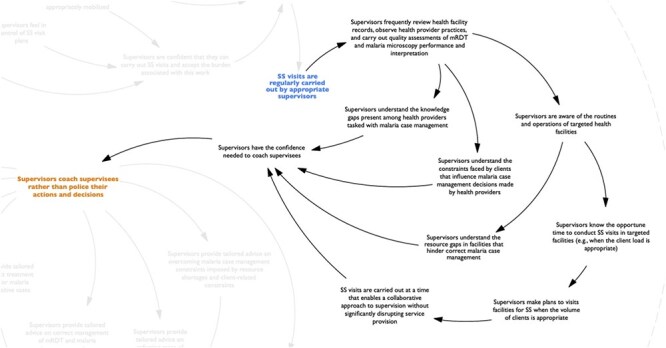
Map of the program pathway (second section).

**Figure 4.**
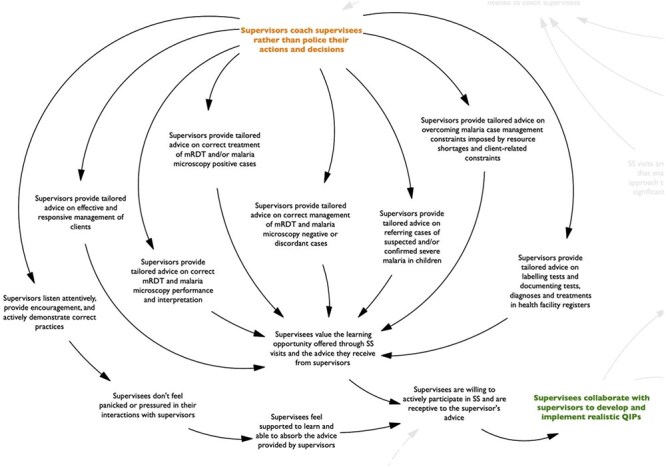
Map of the program pathway (third section).

**Figure 5.**
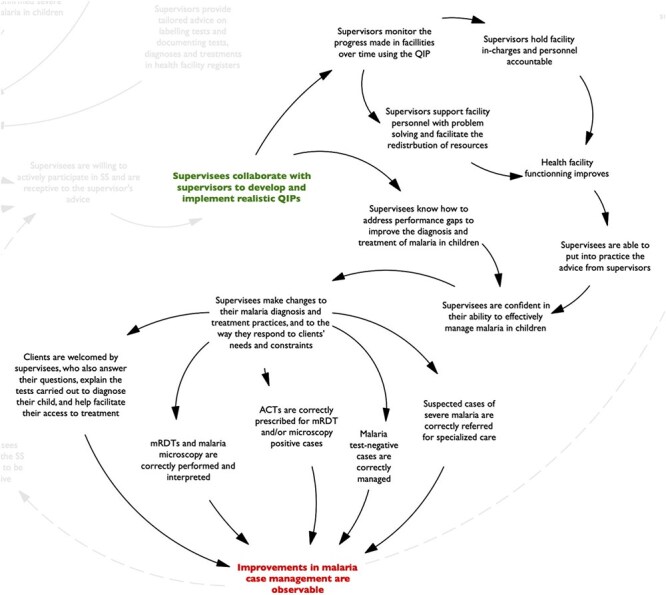
Map of the program pathway (fourth section).

**Figure 6.**
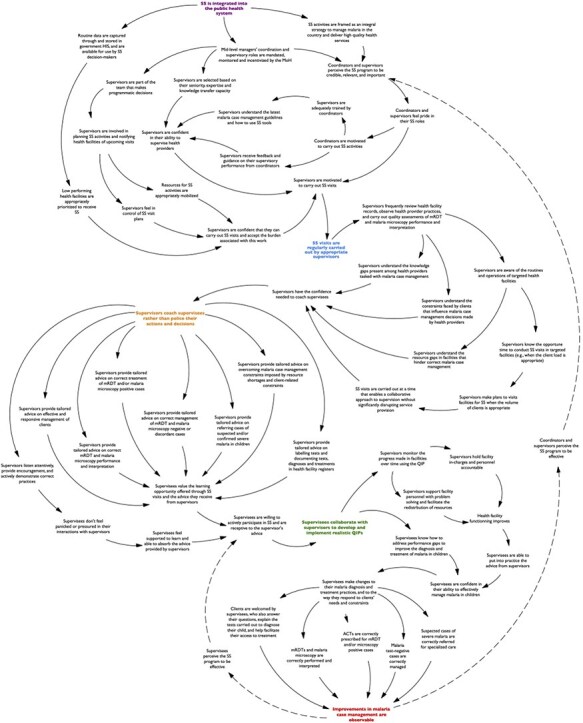
Map of the program pathway (full map).

As mapped in Figs 2 to 6, the pathway through which the mechanisms identified in the RPT drove program outcomes was defined by: (i) the extent to which the program was integrated into the public health system; (ii) the frequency with which SS visits were carried out by appropriate supervisors; (iii) the degree to which supervisors coached, rather than policed, supervisees; and (iv) the level of collaboration achieved between supervisees and supervisors in developing and implementing quality improvement plans.

Key elements in color.

Key elements in color.

Key elements in color.

Key elements in color.

Key elements in color and feedback channels denoted by dashed lines.

First, the program’s ability to produce desired outcomes was largely determined by the extent to which the program was integrated into the public health system (Fig. 2). According to the study’s respondents, this integration ensured that SS coordinators and supervisors were accountable to the MoH, and that their activities aligned with government structures, policies, regulations, and incentives. This alignment enhanced the program’s credibility, relevance, and importance, and also enabling supervisors to be part of the planning of SS activities and facilitating the mobilization of information and resources for the implementation of these activities. Together, these factors dictated the extent to which SS visits were regularly carried out by appropriate supervisors because they shaped: (i) the attitude of coordinators and supervisors towards the program; (ii) the supervisors’ belief that they possessed the expertise, skills, and resources needed to perform the actions associated with their role; and (iii) the burden that supervisors associated with carrying out SS visits.

First of all, [supervisors] will not be able to do the supervision as they had planned if the resources are not there, they will probably delay them going or they will not go at all, but the lack of resources also delays the monitoring of the gaps that exist… so it demoralizes [supervisors] to work. But if he knows the resources are there, he is motivated to work. (Coordinator 02, Round 2, Tanzania)

Because when we go to a [health facility for supervision], we are confident that going there will bring something more to the person we are going to see in the field…Otherwise, if we don’t bring him anything more, it means that it’s useless to go, to continue. (Supervisor 08, Round 2, Benin)

Second, respondents shared that when supervisors carried out SS visits regularly, the quality of each visit improved because the supervisors had more opportunities to review health facility records, observe malaria diagnosis and treatment practices performed by health providers, and conduct quality assessments of mRDT and malaria microscopy performance and interpretation (Fig. 3). These opportunities enabled supervisors to acquire a better understanding of the health providers’ knowledge gaps, the constraints faced by clients that influence health providers’ decision-making, and the resource gaps, routines, and operations of targeted health facilities. This understanding determined the degree to which supervisors adopted a coaching approach to supervision, instead of a more authoritative stance, because it provided them with the confidence to do so.

…we should not take too long [between visits]. You find [a supervisor] who after even six months still has not done [a SS visit]. When you are told to come, you start reviewing again “what am I going to do”, with little opportunity to practice. (Supervisor 17, Round 2, Tanzania)

When we have the habit of carrying out [SS] like this for health providers at their point of care, it allows us - who are at the intermediate level - to ensure that the protocols are really respected. Does the provider have all the protocols at his disposal? Does he understand? … What difficulties is he experiencing and how can we help him to resolve his problems? And when we follow him like this, he gradually feels supported, it also allows him to improve the quality of care he offers to the population. (Coordinator 01, Round 2, Benin)

Third, according to respondents, when supervisors adopted a coaching approach during SS visits, they typically exhibited a range of supportive behaviors (Fig. 4). These behaviors included active listening, providing encouragement, and modeling best practices. They also included offering tailored advice on (i) responsive management of clients; (ii) correct mRDT and malaria microscopy performance and interpretation; (iii) correct treatment of cases based on diagnostic results; (iv) appropriate referral of suspected or confirmed severe malaria cases; (v) correct documentation of tests and diagnoses in health facility information systems; and (vi) solutions to case management challenges imposed by resource shortages and client-related constraints. In the studied contexts where not all health providers tasked with malaria case management had the necessary training for their role and where they experienced resource and client-related constraints that affected their ability to adhere to guidelines, these supportive behaviors largely determined the supervisees’ willingness to engage in SS visits and their receptiveness to the guidance provided by supervisors. This is because the supervisors’ behaviors shaped the value that supervisees attached to SS visits as learning opportunities and influenced the amount of pressure they attributed to interacting with supervisors. Ultimately, these factors determined the level of collaboration that was achieved between supervisees and supervisors in developing and implementing quality improvement plans.

Well, the supervisors who come reassure us before starting so as not to feel panicked and I think they don’t put too much pressure on us, we collaborate well. They treat us as colleagues and tell us to reassure ourselves…they don’t pressure us, so I think there’s a good relationship between us. (Supervisee 11, Round 2, Benin)

It’s true that when people go to supervision, the health [providers] are often not as collaborative… They think…’you’ve come to disturb them…That’s why we say, the supervisor must be a good communicator…Someone social who must take the supervisee as himself…Share with him. (Designer 01, Round 1, Benin)

Because [the supervisor] is already becoming a friend, it becomes very easy to receive what he has brought to me, so it really makes it easier for us to follow what he has directed us to do, so in the end we reach our goals. (Supervisee 15, Round 2, Tanzania)

Fourth, when supervisees and supervisors collaborated to develop and implement quality improvement plans, the supervisees gained a better understanding of the actions needed at both the individual and systemic levels to address performance gaps (Fig. 5). This collaboration also enhanced the supervisors’ ability to monitor the quality improvement plan’s implementation, foster accountability among facility personnel, and support problem solving as needed between SS visits (e.g. working with health facility in-charges to address shortages of essential resources). In the studied context, this collaborative effort nurtured a culture of quality improvement within targeted facilities, which provided supervisees with the support needed to implement the actions recommended by their supervisors. Respondents highlighted that when this pathway was actualized, it culminated in observable improvements to the facility-based management of malaria in children, because supervisees gained confidence in their ability to effectively implement their supervisors’ guidance and were able to make positive changes to their malaria diagnosis and treatment practices.

…we say two-way traffic because you have a discussion and… [the health providers] themselves have to make an action plan. If you make it that way, its implementation becomes easier and after that we review, and every supervisor of the facility does a close monitoring. (Supervisor 02, Round 2, Tanzania)

When [supervisors] finish [the visit], together we…go for the debriefing session. So, they tell us the strong points, and the weak points. What we need to improve….and after that [our facility in-charge] decides to have a staff meeting. So, together we decide what to do about what was said to be weak. (Supervisee 01, Round 2, Benin)

…the knowledge we get [through SS] reassures you and you have the courage to implement what [the supervisor] told you…Because you are confident that you are a good doctor in this aspect, you even feel happy at work. That’s why I think it’s the education they give us and follow us regularly that gives us confidence that we are capable. (Supervisee 15, Round 2, Tanzania)

Furthermore, within this pathway, a notable feedback loop (a feedback loop refers to a causal relationship in which the result of a process impacts the input of that same process) was identified (dashed lines in Fig. 6). That is, according to respondents, as actors involved in SS programs observed its effect (or lack thereof) on malaria case management practices and outcomes, their perception of the programs’ effectiveness changed accordingly. As illustrated by the dashed lines in Fig. 2, this shift in perception not only influenced the pride that SS coordinators and supervisors derived from their roles, but also affected the supervisees’ motivation to engage in SS and their receptivity to the advice offered by supervisors. These changes, in turn, triggered a chain reaction among the interconnected factors within the pathway, ultimately reinforcing the program’s outcomes and the actors’ evolving perception of its effectiveness.

…what will increase the motivation of supervisors in this case is that if you realize that the supervisee is following exactly what you tell him, you can only be motivated to go often to him to see how his work is progressing… (Coordinator 05, Round 2, Benin)

…the responsibility given to [the supervisor] is serious…so they must do the work with great enthusiasm so that in the end the expected results will be produced… it will be something that the supervisor cannot be proud of if within the area the facility has a low performance. (Supervisor 06, Round 2, Tanzania)

And when you put [the supervisor’s advice] into practice, you realize that it’s good, that it helps you. It’s on an intellectual level. That’s what made many of us accept [the supervisors’] advice and coaching and we work with that. (Supervisee 14, Round 2, Benin)

## Discussion

In this study, realist evaluations were conducted in Tanzania and Benin and combined to formulate a program theory, which explains how, why, and under what circumstances SS programs work to improve the facility-based management of uncomplicated malaria in children <5 years of age. As such, this study addresses a critical knowledge gap in SS programming by providing valuable insights into how different contexts and components of SS programs interact to shape the pathway towards program outcomes. In doing so, it offers a deeper understanding of the mechanisms driving this pathway, which can help inform the design and implementation of SS programs Moreover, given similarities between SS programs for malaria case management and those for other primary healthcare services, the findings from this study are likely to have broader relevance for SS programs at improving health service delivery more generally.

In the studied contexts, SS programs designed to improve malaria case management practices were found to be driven by three mechanisms: the affective attitude, self-efficacy, and burden of the program perceived by its main actors. These mechanisms align with several constructs of health intervention acceptability put forth by Sekhon et al. ( 2017) and thereby underscore the importance of aligning the program’s design with the perspectives and needs of its main actors for effective implementation. These findings reinforce the idea that a thorough understanding of the mechanistic properties of SS, as Worges et al. state “that cannot be captured through supervision checklists alone” but that likely determine the impact of SS programs, can greatly benefit program designers and implementers (Worges et al. 2018). In this study, the mechanistic properties of SS programs were uncovered through two rounds of data collection and the development of an IPT and RPT. This method proved advantageous as it allowed for the refinement and expansion of foundational insights. For instance, while the IPT hypothesized that the perceptions of the main actors within SS programs likely played a central role in the programs’ effectiveness, the mechanisms underpinning this effectiveness were only discerned by revising the IPT through a second round of data collection, analysis, and synthesis.

Moreover, by mapping the pathway through which these mechanisms drove program outcomes in the studied contexts, the underlying dynamics of the SS programs were identified. Although pathway or mechanism mapping has been used in implementation science research (Geng et al. 2022, Kilbourne et al. 2023), its application to realist evaluation is novel and merits further consideration given its ability to expand upon a program theory. In this study, the program pathway map revealed that the ability of SS programs to improve malaria case management practices was defined by: (i) the extent to which the program was integrated into the public health system; (ii) the frequency with which SS visits were carried out by appropriate supervisors; (iii) the degree to which supervisors coached, rather than policed, supervisees; and (iv) the level of collaboration achieved between supervisees and supervisors in developing and implementing quality improvement plans. Additionally, within this pathway, a feedback loop was identified, centered on the interrelationship between the program’s outcomes and perceived effectiveness. While there may be other feedback loops within the pathway that were not uncovered by this study, this loop was notable due to its implication for the sustainability of SS programs That is, the reinforcing effect of this loop on the program actors’ perceptions, emotional engagement, motivation, and receptiveness is likely to determine the longer-term engagement and commitment among these actors, consequently either positively or negatively affecting the program’s sustainability.

These dynamics provide insights into many of the SS program components that have been identified in other studies as necessary for improving malaria case management. For example, based on lessons from SS programs in nine countries, Eliades et al. note the importance of incorporating competency criteria for supervisors, technical and supervisory skills training and periodic monitoring of supervisors, and clear criteria for the selection of facilities into the design of SS programs (Eliades et al. 2019a). Also, findings from a more recent independent evaluation of the OTSS approach to quality assurance in malaria service delivery emphasize the significance of a supervision environment characterized by openness and mutual learning in achieving intended program outcomes (Tropical Health 2023).

Overall, these findings are likely to be applicable across a wide range of settings, as the contexts anchoring the program theory are common to many malaria-endemic countries. By scrutinizing the interplay among program contexts, components, and actors’ responses, the theory offers an alternative or complementary perspective to the more commonly utilized successional logic models, such as a theory of change. Consequently, decision-makers can use the program theory as a guide to uncover weaknesses in ongoing programs, understand their downstream impacts, and identify avenues for improvement. To do so, they may wish to consider employing some of the methods used in this study. However, for programs seeking a simpler and less resource-intensive approach to generating insights for informed decision-making, developing routine monitoring indicators that capture the types of contexts and mechanisms described in this study could offer an effective alternative.

Finally, research to investigate the mechanisms that drive the effectiveness of SS programs to improve malaria case management in other contexts would serve to cultivate a broader understanding of these programs. This is specifically important given that, as the findings from this study and others highlight, different approaches to SS are likely to be necessary depending on the program’s contexts (Alombah et al., 2019, Tropical Health 2023). For instance, understanding program mechanisms in contexts where Ministry of Health driven SS programs are not supported by external partners, where supervisors are embedded in health facilities instead of being mid-level managers in the public health system, or where health facilities are consistently well staffed and resourced would be beneficial.

## Limitations

When interpreting the findings from this study, it is important to note that they stem from the perspectives of individuals involved in the design and implementation of SS programs in Tanzania and Benin. These perspectives should not be treated as objective measures of the programs’ functioning. Instead, they represent the respondents’ understanding of their experiences, upon which knowledge was constructed, and a theory was developed. Additionally, it is plausible that the findings were influenced by the perspectives and biases of the researchers who led the study. However, the inclusion of diverse perspectives from a wide array of study participants, coupled with the adoption of a team-based approach to analysis likely served to mitigate potential validity threats.

## Conclusion

This study used realist evaluation to develop a theory based on stakeholders’ perspectives that explains how, why, and under what circumstances SS programs can improve the health facility-based management of uncomplicated malaria in children <5 years of age. The study established that, in specific but common contexts, the outcomes of SS programs designed to improve malaria case management practices are driven by the affective attitude, self-efficacy, and burden of the program perceived by its main actors. The study also demonstrated why the perceived effectiveness of the program is likely to be central to its sustainability. Collectively, these findings underscore the importance of considering the cognitive and emotional responses of program actors throughout the design, implementation, monitoring, and evaluation of SS programs. Although these insights are likely to be applicable to malaria-endemic settings with shared contexts, further research should explore the mechanisms driving SS program effectiveness in different contexts to further address existing knowledge gaps.

## Supplementary Material

czaf020_Supp

## Data Availability

The data underlying this article will be shared on reasonable request to the corresponding author.

## Reflexivity Statement

The authors include 5 females and 14 males and span multiple levels of seniority. Among the 19 authors, 11 identify primarily as researchers, 4 as policymakers in Tanzania and Benin, and 4 as representatives of US federal agencies in Tanzania and Benin. Among the 11 researchers, 8 are based in Tanzania and Benin with expertise in child health and malaria, 3 are based in the UK with expertise in the evaluation of complex health interventions and malaria, and all have experience conducting mixed methods and realist evaluations in sub-Saharan Africa.
