# Supplementary material for: How, why, and under what circumstances can supportive supervision programs improve malaria case management? A realist program theory
Source: Health Policy Plan. 2025 Mar 26;40(6):600–12. doi: 10.1093/heapol/czaf020 (PMC12160800; doi:10.1093/heapol/czaf020)
Supplement: czaf020_Supp [file czaf020_supp.zip › Revised supplementary materials.docx]

## **SUPPLEMENTARY MATERIALS**

**Initial program theory**

1. In a context where the Ministry of Health prioritizes the improvement of malaria case management **(C),** if the program is integrated into the public health system **(I),** then national and sub-national stakeholders **(A)** will feel ownership of the program **(M),** so they will take action to support its adoption **(O)**.
2. In a context where mid-level managers serve as supportive supervision (SS) coordinators and supervisors **(C),** if the SS program provides clear and concise tools and comprehensive training to supervisors **(I),** then the supervisors **(A)** will feel confident in their ability, and be willing, to carry out the responsibilities required by their roles **(M),** so the program will be implemented according to plan **(O).**
3. In a context where health providers struggle to consistently adhere to malaria diagnosis and treatment guidelines **(C)**, if the SS program encourages supervisors to build a good rapport with supervisees and to work with supervisees to resolve knowledge and performance gaps **(I)**, then the supervisees **(A)** will have a favorable outlook on SS **(M)**, so they will actively engage in SS visits **(O)**.

N.B.: C - context; I - intervention; A - actor; M - mechanism; O - outcome

**Table I.** Summary of findings from two rounds of IDIs conducted in Tanzania and Benin (N=218)

| **Core findings** | **Tanzania** | **Benin** |
| --- | --- | --- |
| **Contexts** | | |
| Prevailing contexts | - Improving the management of malaria in children under five years of age was a national priority and championed by the MoH. - Financial and technical support for SS were provided, in part, by an external partner. - Malaria-related services for children under five years of age were free of charge to the client. - Mid-level managers (at the regional and council levels) had heavy workloads and limited transport but were tasked with serving as SS coordinators and supervisors. - Some health facilities were understaffed and experienced considerable staff turnover. - Not all health providers involved in malaria case management received the necessary training to fulfil this role. - Health providers working in health facilities had limited opportunities to refresh their knowledge and were accustomed to inspection-style supervision. - Some health facilities occasionally experienced shortages of key malaria diagnosis and treatment supplies. - The economic status of many communities was low. - Some clients (parents or guardians of febrile children) hesitated to seek services in health facilities where they perceived the quality of care to be poor. - In some communities, cultural beliefs and misinformation about malaria influenced care seeking behaviors and resulted in attempts to influence the service provision process (e.g., refusal of malaria tests due to fear, or requests for ACTs without tests). | - Improving the management of malaria in children under five years of age was a national priority and championed by the MoH. - Financial and technical support for SS were provided, in part, by an external partner. - Coordinators and supervisors believed that SS workplans and budgets could be integrated into the public health systems. - The adherence to a hierarchical structure, determined by factors like education, experience, and one's position within the health administration pyramid, was ingrained among individuals working within the health system. Authoritative top-down administrative approaches were also normalized within the health system. - Mid-level managers (at the zone and department levels) reported having heavy workloads and a shortage of transport. The number of mid-level managers who had the expertise and experience required to serve as SS supervisors was limited. - Malaria-related services were provided in health facilities every day, and most health facilities had the capability to perform malaria microscopy. - Most, but not all, providers received some form of integrated management of childhood illness training, which provided basic knowledge on correct malaria case management. - Health facilities and laboratories normally had the supplies and materials needed to correctly diagnose and treat malaria in children under five years of age. However, mRDTs were at times temporarily stocked out in facilities with high client loads. - Many clients reportedly had low purchasing power and some attempted to influence the service provision process when seeking care. - Cultural norms, concerns about blood tests, and trust in traditional medicine influenced care-seeking behaviors in some communities. |
| **Program components** | | |
| Facility targeting | - All facilities were eligible to receive SS. - Poor-performing facilities were prioritized. - Effective facility targeting relied on accessible high-quality data. Supervisors trusted data they collected and that was managed by the government. - A targeted approach was seen as optimizing the use of resources, but potentially resulting in missing facilities that could benefit from visits. | - All facilities were eligible to receive SS. - Priority was given to facilities that were not recently supervised and poorer-performing facilities when feasible. - Decision-makers favored readily available routine dataset indicators over data from previous SS visits. - A targeted approach was seen as optimizing the use of resources. |
| Frequency of SS visits | - Quarterly visits were believed to allow enough time for observable improvements. - Irregular visits were believed to result in health providers reverting to old habits. - Supervisors faced challenges in regularly visiting health facilities due to budget, time, and transportation constraints. - Visiting all facilities regularly was not feasible for supervisors. | - Quarterly health facility visits, and bi-annual laboratory visits were believed to allow enough time for observable improvements. - Prompt follow-up visits were conducted to sustain progress and prevent regressions. - Supervisors faced challenges in regularly visiting health facilities due to resource constraints, including limited transportation and delays in reimbursements. |
| Selection of supervisors | - Limited availability of suitable supervisors was a common issue. - Mid-level managers were often chosen as supervisors given their technical knowledge and knowledge of health facilities. - Some supervisors lacked expertise in specific areas, such as malaria microscopy. | - Limited availability of suitable supervisors was an issue at times. - Most supervisors had the required technical knowledge and status to earn respect from supervisees, but some were from outside the facilities’ catchment areas, neared retirement, or lacked recent service delivery experience, which garnered less respect. |
| Training and supervision of supervisors | - Supervisors were expected to be trained for their role, but not all received training. - Some supervisors missed the initial training and relied on peer learning. - Training helped supervisors understand malaria case management and use the SS tools effectively. - Feedback on supervisor performance came from various sources (e.g., regional malaria coordinators and health management teams) and focused on the SS tool’s use. | - Supervisors were expected to be trained for their role, but not all received training. - Training helped supervisors refresh their knowledge of malaria case management guidelines, understand SS tools, and learn effective coaching techniques. Training was especially valuable for supervisors who were not recently involved in service delivery. - Health facility-focused supervisors received regular performance feedback from departmental actors and laboratory-focused supervisors had little performance feedback. |
| Planning of SS visits | - Notifying health facilities in advance of SS visits was seen as important to avoid surprising them and allow health providers to prepare. - The health providers' schedules and client load at health facilities were crucial factors in visit planning. - Conducting SS visits when facilities had fewer clients facilitated supervisees’ participation in SS activities, and the observations of case management practices. | - Health facility-focused supervisors actively collaborated with department coordinators to plan SS visits and laboratory-focused supervisors primarily executed plans developed by national-level actors. - Notifying health facilities in advance of SS visits was seen as important to avoid surprising them and to allow health providers to prepare. Supervisees were not all informed about upcoming visits, despite the health facility in-charge being notified. |
| SS tool | - The tool's limitations affected the frequency and quality of visits, frustrating supervisors. - Concerns were raised about outdated or irrelevant indicators in the tool. - Extensive checklists that lacked integration with other supervision programs were considered time-consuming. - Client interviews were uncommon despite their inclusion in the tools. - The electronic tool was seen as making SS visits more efficient than paper-based tools. However, the use of smartphones to collect data posed notable challenges. | - Supervisors highly valued the SS tools. Initially, there were concerns about the tools' length, but revisions made them suitable for most supervisors. - Suggestions for enhancing the tools included revisions to better consider client and community perspectives. - The tools were paper-based, and no respondents advanced the idea of using electronic tools. |
| Selection of supervisees | - There was no deliberate selection process for addressing individual performance gaps. - Availability on the day of the SS visit was the de facto criterion for selection of health providers. - Some viewed the absence of guidelines positively, as it provided equal opportunities for all health providers to be supervised. Others were concerned that it led to some health providers being overlooked. - Some thought that intentional selection of poorer performing health providers could optimize resource use and improve the quality of malaria case management. | - There was no deliberate selection process for addressing individual performance gaps. - Availability on the day of the SS visit was the de facto criterion for selection of health providers. This approach was seen as a weakness, resulting in some individuals receiving multiple supervisions while others were overlooked. - Concerns were raised that teams might be strategically scheduling stronger performers during visits to avoid negative reports on overall laboratory performance. - Some nurses' aides received SS, but others didn’t due to concerns about policy misalignment. |
| Observation of practices | - Supervisors observed and assessed adherence to diagnosis and treatment guidelines, and provider-client interactions. - Observations were believed to increase the supervisors’ awareness of facility challenges. - Feedback through hypothetical case discussions, used in the absence of real cases, was considered less effective than direct observations. | - Supervisors observed and assessed adherence to diagnosis and treatment guidelines, and provider-client interactions. - Direct observations helped supervisors understand how health providers managed specific situations, such as when clients expressed treatment preferences or constraints. - Feedback through hypothetical case discussions, used in the absence of real cases, was considered less effective than direct observations. |
| Performance of quality assessments | - Supervisors examined mRDTs and blood slides (malaria microscopy) during SS visits to assess test performance, interpretation, labeling, and record-keeping. - Effective assessments depended on the proper storage and cataloging of blood slides and mRDTs. | - Supervisors conducted quality assessments of malaria diagnostic tests (mRDTs and malaria microscopy) performed by health providers. - Supervisors also independently examined slides, comparing their interpretations with those of the supervisee to ensure consistent and accurate result interpretations. |
| Review of facility records | - Supervisors reviewed facility records to examine compliance with reporting guidelines, understand the use of malaria tests and treatments, and identify resource gaps preventing adherence to malaria case management guidelines. - As mid-level managers, supervisors were considered well-placed within the health system to address resource gaps in facilities. | - Supervisors reviewed facility records to examine compliance with reporting guidelines, understand the use of malaria tests and treatments, and identify resource gaps preventing adherence to malaria case management guidelines. - As mid-level managers, supervisors were considered well-placed within the health system to address resource gaps in facilities. |
| Provision of advice on appropriate practices | - Supervisees received advice on diagnosing and treating febrile children and on managing clients more generally. - They were reminded to adhere to mRDT and malaria microscopy protocols, and treatment guidelines; how to recognize malaria symptoms, including signs of severe disease; how to build trust among clients in diagnostic tests and to educate clients on the proper administration of treatments; and how to properly manage, monitor, and procure malaria-related supplies and equipment. | - Supervisees received advice on accurate diagnosis of malaria cases, correct treatment of uncomplicated malaria and referral for suspected severe malaria, and effective client management. - They were reminded how to address clients’ financial constraints and how to explain diagnostic and treatment steps thoroughly to address clients’ concerns; and were given advice on how to diagnose and treat febrile children in the absence of essential materials or supplies. |
| Development and monitoring of quality improvement plans (QIPs) | - QIP development during SS visits was common practice. - Some supervisors developed QIPs with supervisees, which helped supervisees understand individual practice and systemic gaps, as well as solutions. - Sharing the QIP with all personnel in the facility to facilitate facility-wide improvements was believed to be important. | - At the end of SS visit, health facility-focused supervisors developed ‘problem-solving’ plans with supervisees, while laboratory-focused supervisors authored reports and shared them with supervisees. - To ensure implementation of the outlined actions, supervisors used telecommunication to keep facility personnel accountable and provide support between SS visits. |
| **Key factors influencing program outcomes** | | |
| Supervisors are motivated to carry out SS activities | - Supervisors’ motivation was influenced by 1) their perception that SS activities could be executed as planned, 2) effective teamwork, 3) timely disbursement of funds and mobilization of resources, and 4) their belief that SS aligned with national priorities. - Recognition of achievements enhanced supervisors’ pride and thereby their motivation to carry out their SS role. - Integration of SS with other routine supervisory activities was not universally viewed as beneficial, as it could divert attention and compromise the program’s effectiveness. | - Supervisors’ motivation was influenced by the sense of pride they derived from their supervisory responsibilities. Witnessing the impact of their efforts, such as improved client care, was a significant source of pride. - Active participation from supervisees during SS visits and recognition of their achievements by their superiors in the health system hierarchy also motivated supervisors. - Although remuneration was a motivator for some, it was not considered a driver by all. Timely reimbursement of expenses incurred during SS visits was crucial. - Integrating SS with other routine supervision tasks was not universally seen as beneficial. |
| Supervisors are confident in their capacity to effectively implement SS activities | - Supervisors’ confidence in fulfilling their role was linked to their knowledge of the latest malaria case management guidelines and expertise in their specific area of oversight. - Training and clear expectations relating to their role played an important role in expanding supervisors' confidence. - Opportunities to learn about performance gaps in health facilities were considered crucial. | - Supervisors’ confidence in conducting SS activities was linked to their control over the planning these activities, their ability to work within a team, and their relative expertise compared to supervisees. - Trust and respect from supervisees and appointing authorities also enhanced supervisors' confidence. - Opportunities to stay updated on malaria guidelines were considered essential. |
| Supervisees feel supported to learn | - Supervisees felt supported to learn when supervisors were friendly and helpful. - Supervisees appreciated supervisors who dedicated ample time to mentoring them. - A participatory approach to SS, involving supervisees in problem-solving made supervisees more receptive to advice. - Supervisees preferred different supervisors for repeat visits to benefit from diverse experts. - Supervisors’ heavy workload was believed to undermine the quality of SS visits. | - Supervisees felt supported to learn when supervisors were friendly, helpful, and humble. - A collaborative and encouraging approach to SS visits reduced supervisees’ stress and created a conducive learning environment, especially when supervisees’ authority as health providers was respected by supervisors in front of clients. - Coaching was challenging in facilities with a high volume of clients as supervisees had to balance collaborating with supervisors and managing their clients’ needs. - Supervisees preferred different supervisors for repeat visits to benefit from diverse experts and to avoid familiarity-related attention failures. |
| Supervisees are willing to actively participate in SS visits | - Supervisees’ willingness to actively participate in SS visits was influenced by their perceived value of these visits. - Informing supervisees of upcoming SS visits helped them mentally prepare. - Supervisees were more willing to engage in SS visits when they were coached by supervisors. - Previously benefiting from SS visits increased willingness to participate in future visits. - Supervisors' knowledge and understanding of service constraints determined the value that supervisees attributed to the supervisors’ advice. | - Supervisees’ willingness to participate in SS visits was tied to the belief that they could gain new knowledge from supervisors. - Supervisors with recent field experience and the necessary status were more likely to be seen as knowledgeable. - Supervisees preferred to be coached by supervisors (rather than policed) and this influenced their willingness to engage in SS visits. - In understaffed facilities, supervisees recognized the value of participating in SS when visits did not disrupt service provision. |
| Supervisees are willing and feel able to adopt the advice from supervisors | - Supervisees’ willingness to adopt the supervisors' advice was influenced by their perception of the value of the advice. - A good rapport with supervisors, ongoing performance monitoring and actionable guidance from supervisors that considered service delivery constraints, encouraged adoption. - Availability of essential supplies and resources was crucial for implementation of advice. | - Supervisees were more willing to adopt the supervisors' advice when they trusted its accuracy and effectiveness and when their supervisor had more seniority than them. - Concerns about reputation and avoiding embarrassment in front of the community, supervisors, or colleagues motivated supervisees to adopt the supervisors’ advice. - Ongoing performance monitoring and actionable guidance from supervisors, that considered service delivery constraints, encouraged adoption. - Availability of essential supplies and resources was crucial for adoption of advice. |
| Supervisees are confident in their capacity to improve their performance | - Supervisee confidence was tied to their understanding of correct case management practices, which was bolstered by timely and tailored advice from supervisors. - A supportive learning environment and involvement in QIP development and implementation affected their confidence. - Access to key supplies influenced supervisees’ ability and confidence. | - Supervisees felt confident when they consistently received constructive feedback and encouragement from supervisors who observed their practices. - Their confidence was reinforced when they were actively involved in developing problem-solving plans and when they observed tangible improvements in their performance. |
| **Reported outcomes – Health provider performance** | | |
| Increase in test-based diagnoses | - SS visits underscored the importance of test-based malaria diagnoses, influencing providers' practices and their communication with clients about the value of tests. - Consequently, clients' resistance to testing reportedly diminished, test-based diagnoses reportedly increased (decreasing clinical diagnoses of malaria) in health facilities. | - Ongoing SS visits reportedly led to an increase in test-based diagnoses for malaria because health providers were more knowledgeable about the latest malaria case management guidelines and because regular assessments by supervisors left little room for protocol deviations to go unnoticed. |
| Improvement in the quality of mRDT and malaria microscopy performance and interpretation | - Supervisors' observations and feedback reportedly led to notable improvements in the quality of mRDT performance and interpretation. - Consequently, supervisees gained confidence in their abilities and trust in test reliability, reducing their hesitance to use tests. - Supervisors believed SS enhanced the quality of malaria microscopy performance and interpretation. | - SS visits reportedly led to notable improvements in the quality of mRDT performance and interpretation. Unnecessary mRDT utilization reportedly decreased, and internal and external quality control of malaria microscopy improved. - Health providers reportedly gained increased trust in test results, leading to a greater willingness to use mRDTs and request microscopy for diagnosing febrile children. - Low utilization of confirmatory microscopy in some facilities was attributed to laboratory closures on weekends and clients' inability to afford the service. |
| Improvement in the appropriate treatment of malaria test-positive and test-negative cases | - SS was perceived to have positively impacted the appropriate treatment of malaria test-positive cases. - Supervisors' guidance on prescribing practices, accurate dosages for children, client education, and maintaining records, even during ACT stock-outs, was highly valued by health providers. | - Children under five years who tested positive for malaria were reportedly more likely to receive correct ACT prescriptions as a result of SS and malaria test-negative cases receiving ACTs reportedly decreased in most health facilities that received SS visits. - Health providers were said to adhere to recommended weight-dose ratios and to refer cases of severe malaria more frequently for appropriate management. |
| **Reported outcomes – Health facility performance** | | |
| Culture of quality improvement in health facilities | - The benefits of SS reportedly extended beyond supervisees, as sharing the QIP with all facility personnel was perceived to improve the performance of unsupervised health providers as well. | - SS visits reportedly fostered a culture of quality improvement in regularly visited facilities as lessons learned through SS were disseminated to all facility personnel and accountability and teamwork were promoted to enhance the quality of care. |
| Improved records | - SS reportedly improved record-keeping and test labelling through record monitoring. | - The quality of record keeping reportedly improved, despite some retroactive revisions. |
| Decrease in resource gaps | - Regular SS visits were seen to positively impact the availability of key supplies, (e.g., mRDTs and reagents) by providing opportunities to identify and address resource gaps. | - Improved record keeping was believed to have led to more accurate supply estimates and fewer resource gaps. |

**Table II.** Summary of findings from the facility audits conducted in Benin (N=4)

|  | **Facility A** | **Facility B** | **Facility C** | **Facility D** |
| --- | --- | --- | --- | --- |
| **Facility functioning** | | | | |
| Highest professional qualification of the head of facility | Doctor | Nurse | Doctor | Nurse |
| Most frequented time | Weekday mornings | Weekday mornings | Weekday mornings | Weekday evenings |
| Located near an urban area | No | No | No | Yes |
| Use of computerized stock management system | No | Yes | No | Yes |
| Medication dispensing hub in facility | Facility pharmacy & maternity ward | Facility pharmacy | Facility pharmacy | Facility pharmacy |
| Availability of microscopy services in the facility | Yes | Yes | Yes | Yes |
| - Frequency of microscopy availability | 5 days/week | 7 days/week | 5 days/week | 5 days/week |
| - Number of functioning microscopes | 1 | 1 | 1 | 1 |
| Availability of mRDT services in the facility | Yes | Yes | Yes | Yes |
| - Point of care for mRDTs in the facility | Nurses’ office | Nurses’ office | Nurses’ office | Facility entrance |
| **Key personnel** | | | | |
| Doctor | 1 | 1 | 1 | 0 |
| Nurse | 4 | 6 | 7 | 7 |
| Midwife | 4 | 2 | 3 | 4 |
| Nurses’ aide (*Aide-soignant*) | 9 | 6 | 7 | 7 |
| Laboratory technician | 1 | 2 | 1 | 1 |
| Pharmacy clerk | 2 | 2 | 2 | 3 |
| **Availability of medicines** | | | | |
| Artemether/Lumefantrine | Available and valid | Available and valid | Available but stocked out | Available and valid |
| Artesunate injectable | Available and valid | Available and valid | Stocked out for 6 months | Stocked out for 6 months |
| Quinine injectable | Never available | Available and valid | Never available | Stocked out for 6 months |
| **Average cost to clients of services** | | | | |
| Consultation for a febrile child | 2,000 CFA ($3.40 USD) | 500 CFA ($0.80 USD) | 600 CFA ($1.00 USD) | 400 CFA ($0.70 USD) |
| mRDT | Free | Free | Free | Free |
| Microscopy | 1,500 CFA ($2.50 USD) | 500 CFA ($0.80 USD) | 1,000 CFA ($1.70 USD) | 1,500 CFA ($2.50 USD) |
| ACTs | 2,000 CFA ($3.40 USD) | 300 CFA ($0.50 USD) | 1,500 CFA ($2.50 USD) | 1,000 CFA ($1.70 USD) |
| **SS visits in general** | | | | |
| Number of SS visits received in the preceding 12 months | 4 | 5* | 4 | 4 |
| Presence of SS coordinators in the facility during SS visits | Yes | Yes | No | No |
| **Latest SS visit** | | | | |
| Advance notice of the SS visit was given to facility personnel | Yes | Yes | Unknown | Yes |
| Direct observations of febrile case management were performed | Yes | Yes | No | Yes |
| A report on the SS visit was written by the supervisor | Yes | Yes | No | Yes |
| Number of providers supervised during the visit | 7 | 6 | 5 | 11 |
| - Nurse | 2 | 1 | 1 | 5 |
| - Midwife | 2 | 1 | 1 | 0 |
| - Laboratory technician | 1 | 1 | 1 | 1 |
| - Nurses’ aide (*Aides soignant*) | 2 | 3 | 2 | 5 |

*Supervisors returned to Facility B a fifth time to compensate for a missed visit during the preceding year.

**Table III.** Summary of core findings from the structured interviews with health providers conducted in Benin (N=12)

|  | | **Facility A** | **Facility B** | **Facility C** | **Facility D** | **Overall %** |
| --- | --- | --- | --- | --- | --- | --- |
| **Providers’ profile** | | | | | | |
| Male | | 2/3 | 2/2 | 2/4 | 3/3 | 75.0% |
| Originally from outside of the health facility’s zone | | 2/3 | 1/2 | 1/4 | 3/3 | 58.3% |
| Highest level of formal education | Primary certificate | 0/3 | 0/2 | 1/4 | 0/3 | 8.3% |
|  | Secondary school | 2/3 | 1/2 | 2/4 | 3/3 | 66.6% |
|  | University 1st Cycle (e.g., Nursing Diploma) | 1/3 | 1/2 | 0/4 | 0/3 | 16.7% |
|  | University 3rd Cycle (e.g., Medical Degree) | 0/3 | 0/2 | 1/4 | 0/3 | 8.3% |
| Have attended any training on IMCI | | 1/3 | 1/2 | 2/4 | 1/3 | 41.7% |
| Have attended any training on malaria diagnosis protocols | | 3/3 | 2/2 | 3/4 | 3/3 | 91.7% |
| Have attended any training on malaria treatment protocols | | 3/3 | 2/2 | 4/4 | 3/3 | 100% |
| Perceive a need for additional training to correctly manage febrile children <5 years | | 3/3 | 1/2 | 3/4 | 3/3 | 83.3% |
| **Providers’ role and workload** | | | | | | |
| Role in the health facility | Physician | 0/3 | 0/2 | 1/4 | 0/3 | 8.3% |
|  | Nurse | 3/3 | 2/2 | 2/4 | 3/3 | 83.3% |
|  | Nurses’ aide (*Aide-soignant*) | 0/3 | 0/2 | 1/4 | 0/3 | 8.3% |
| Duration of service in the health facility | Less than a year | 1/3 | 0/2 | 1/4 | 0/3 | 16.7% |
|  | 1 to 3 years | 1/3 | 2/2 | 0/4 | 1/3 | 33.3% |
|  | More than 3 years | 1/3 | 0/2 | 3/4 | 2/3 | 50.0% |
| Workload perceived as manageable | | 3/3 | 2/2 | 3/4 | 2/3 | 83.3% |
| Frequency of access to supplies and equipment needed to fulfil role | Always have access | 1/3 | 1/2 | 1/4 | 1/3 | 33.3% |
|  | Usually have access, but not always | 2/3 | 1/2 | 3/4 | 2/3 | 66.7% |
| **Routine supervision** | | | | | | |
| Providers who reported having a routine supervisor in the health facility | | 3/3 | 2/2 | 3/4 | 3/3 | 91.7% |
| Frequency of routine supervision received by providers | Daily | 0/3 | 0/2 | 3/3 | 1/3 | 36.4% |
|  | Weekly | 1/3 | 0/2 | 0/3 | 0/3 | 9.1% |
|  | Monthly or less frequently | 1/3 | 2/2 | 0/3 | 2/3 | 45.5% |
|  | When needed | 1/3 | 0/2 | 0/3 | 0/3 | 9.1% |
| Type of routine supervision received by providers in the preceding 6 months | Group supervision | 3/3 | 0/2 | 1/3 | 0/3 | 36.4% |
|  | One-on-one supervision | 0/3 | 2/2 | 2/3 | 2/3 | 54.5% |
|  | Spot checks | 0/3 | 0/2 | 2/3 | 1/3 | 27.3% |
| **Supportive supervision received by providers** | | | | | | |
| Received SS more than once | | 3/3 | 2/2 | 2/4 | 2/3 | 75.0% |
| Number of SS visits received by providers in the last 3 years | 1 visit | 0/3 | 0/2 | 2/4 | 1/3 | 25.0% |
|  | 2 visits | 2/3 | 0/2 | 0/4 | 1/3 | 25.0% |
|  | 3 visits | 0/3 | 1/2 | 0/4 | 0/3 | 8.3% |
|  | 4 visits | 1/3 | 0/2 | 2/4 | 1/3 | 33.3% |
|  | 5 visits | 0/3 | 1/2 | 0/4 | 0/3 | 8.3% |
| Supervised by different SS supervisors (among providers who received at least 2 SS visits) | | 1/3 | 2/2 | 2/2 | 2/2 | 77.8% |
| Reported a preference to be supervised by different SS supervisors each time | | 3/3 | 2/2 | 3/4 | 3/3 | 91.7% |
| Reported experiencing a collaborative interaction with SS supervisors | | 3/3 | 2/2 | 4/4 | 3/3 | 100% |

**Table IV.** Outcomes reported in an audit of 154 febrile cases of children <5 years in Benin (decisions summarized in Table V)

|  | **Facility A** | | **Facility B** | | **Facility C** | | **Facility D** | | **Total** | |
| --- | --- | --- | --- | --- | --- | --- | --- | --- | --- | --- |
|  | **n** | **%** | **n** | **%** | **n** | **%** | **n** | **%** | **n** | **%** |
| **Febrile* cases tested for malaria** | 35 | 100 | 33 | 100 | 48 | 100 | 38 | 100 | 154 | 100 |
| Febrile cases that underwent a mRDT only | 32 | 91.4 | 32 | 97.0 | 47 | 97.9 | 18 | 47.4 | 129 | 83.8 |
| Febrile cases that underwent malaria microscopy only | 0 | 0.0 | 0 | 0.0 | 0 | 0.0 | 3 | 7.9 | 3 | 1.9 |
| Febrile cases that underwent a mRDT and malaria microscopy | 3 | 8.6 | 1 | 3.0 | 1 | 2.1 | 17 | 44.7 | 22 | 14.3 |
| **Malaria test-positive cases** | 11 | 31.4 | 23 | 69.7 | 34 | 70.8 | 21 | 55.3 | 89 | 57.8 |
| Malaria RDT-positive cases | 8 | 72.7 | 22 | 95.7 | 34 | 100 | 11 | 52.4 | 75 | 84.3 |
| Malaria microscopy-positive cases | 3 | 27.3 | 0 | 0.0 | 0 | 0.0 | 7 | 33.3 | 10 | 11.2 |
| Malaria RDT- and microscopy-positive cases | 0 | 0.0 | 1 | 4.3 | 0 | 0.0 | 3 | 14.3 | 4 | 4.5 |
| **Malaria test-positive case diagnosed with uncomplicated malaria** | 3 | 27.3 | 8 | 34.8 | 30 | 88.2 | 16 | 76.2 | 57 | 64.0 |
| Test-positive cases diagnosed with uncomplicated malaria given an ACT | 3 | 100 | 8 | 100 | 30 | 100 | 16 | 100 | 57 | 100 |
| **Malaria test-positive case diagnosed with suspected or confirmed severe malaria** | 8 | 72.7 | 15 | 65.2 | 4 | 11.8 | 5 | 23.8 | 32 | 36.0 |
| Test-positive cases suspected or confirmed severe malaria referred for specialized care | 0 | 0.0 | 11 | 73.3 | 2 | 50.0 | 5 | 100 | 18 | 56.3 |
| Test-positive cases suspected or confirmed severe malaria given an ACT | 1 | 12.5 | 3 | 20.0 | 2 | 50.0 | 0 | 0.0 | 6 | 18.8 |
| Test-positive cases suspected or confirmed severe malaria given artesunate (not referred, no ACT) | 6 | 75.0 | 1 | 6.7 | 0 | 0.0 | 0 | 0.0 | 7 | 21.9 |
| **Malaria test-negative cases** | 24 | 68.6 | 10 | 30.3 | 14 | 29.2 | 17 | 44.7 | 65 | 42.2 |
| Malaria test-negative cases given non-malaria diagnosis | 21 | 87.5 | 10 | 100 | 13 | 92.9 | 16 | 94.1 | 60 | 92.3 |
| Malaria test-negative cases not diagnosed with malaria given an ACT | 0 | 0.0 | 0 | 0.0 | 1 | 7.7 | 0 | 0.0 | 1 | 1.7 |
| Malaria test-negative cases and diagnosed with uncomplicated malaria | 3 | 12.5 | 0 | 0.0 | 1 | 7.1 | 1 | 5.9 | 5 | 7.7 |
| Malaria test-negative cases and diagnosed with uncomplicated malaria given an ACT | 0 | 0.0 | 0 | - | 0 | 0.0 | 1 | 100 | 1 | 20.0 |
| Malaria test-negative cases and diagnosed with severe malaria | 0 | 0.0 | 0 | 0.0 | 0 | 0.0 | 0 | 0.0 | 0 | 0.0 |
| Malaria test-negative cases and diagnosed with severe malaria given an ACT | 0 | - | 0 | - | 0 | - | 0 | - | 0 | - |

*Cases were considered febrile when a fever was reported by a parent/guardian to the health provider and/or confirmed by a health provider in the health facility.

**Table V.** Summary of decisions made by health providers who managed the cases audited in Benin (N=154)

| **Decision** | **Explanation of decision** | **Illustrative quotes from interviewers’ notes** |
| --- | --- | --- |
| Testing febrile children <5 years | - Providers primarily reported requesting mRDTs for children under five years of age on the basis of a fever reported (by a parent/guardian) or confirmed (by a provider) as recommended by their SS supervisors. Providers also reported considering other signs and symptoms in their decision, including vomiting, coughing, diarrhea, convulsions, fatigue, throat ache, abnormal respiratory rate, acute malnutrition, and pallor. Additional factors that reportedly shaped their decision to request an mRDT included (a) the presence of recent rainfall; (b) the information provided by the guardians/parents; (c) consent from parents/guardians for conducting the mRDT; (d) the provider’s experience in managing malaria cases; (e) the provider’s willingness to follow the IMCI flowchart; and (f) the malaria case management training received by providers during their formal studies. - Providers reported requesting malaria microscopy based on the advice received from SS supervisors to corroborate a positive mRDT. However, the presence of a fever in children combined with a negative mRDT also reportedly prompted providers to request malaria microscopy. - The availability of mRDTs and laboratory services in health facilities, and the clients’ ability to pay for laboratory services were also considered by providers. In facility D, three of the 38 febrile cases did not undergo an mRDT due to a temporary stock-out of mRDTs in the health facility (caused by a shipment mix-up). Providers directed these cases to a nearby private laboratory for malaria microscopy. The greater use of malaria microscopy in this facility was linked to its proximity to said laboratory, which remained open on weekends. Also, in facilities A, B, and C, and to a lesser degree in facility D, providers were often unable to follow through with malaria microscopy because clients were unable to afford the cost of this service. | - *“What we learned through supportive supervision is that when a patient comes and presents with a fever, for example, and you do the mRDT and it is negative, go further with testing and see. The reason for which it is necessary to ask for the blood smear is to see if it is not a hidden malaria. Maybe the parasite density is not high yet. That's why our mRDT didn't show, so we have to go a little further.”* - “*The supervisors' advice influenced my decision because without a positive mRDT, there is no prescription for ACTs. And today the mRDT was not available so we waited for the malaria microscopy results before giving a diagnosis and treatment.”* - *“Supportive supervision helps us with everything. Without microscopy confirmation one cannot prescribe a drug to the child. Before we bombarded treatments even though we could have been intoxicating the child. It is thanks to supportive supervision that I am requesting these tests to see more clearly. Before, we closed our eyes and prescribed ACTs. Sometimes it worked, sometimes it didn’t…Thanks to supportive supervision, I am sharp in what I do, and my heart is at ease and clear.”* - *“I did not ask for malaria microscopy because the mother had told me that she did not have the means. I had already made the request and had to cancel it… It was the advice* [from SS supervisors] *that told me that if the mRDT is negative, you must ask for a blood smear. I asked but the patient said that she had no money, so I was stalled.”* |
| Diagnosing and treating malaria test-positive cases in children <5 years | - Providers consistently prescribed ACTs to malaria case-positive children diagnosed with uncomplicated malaria. Providers reportedly considered in this decision both the national malaria case management guidelines and the IMCI flowchart, as well as the advice received from SS supervisors about administering ACTs to test-positive cases. - Also, ACTs were not typically given to malaria test-positive cases by providers if severe malaria was suspected (based on signs and symptoms) or confirmed (based on microscopy results). Providers mainly referred these cases to the nearest services for specialized care (18/32) or administered artesunate (7/32). However, some cases were given ACTs following artesunate injections (6/32). These decisions were reportedly largely motivated by the national malaria guidelines and the guidance received through SS. Exceptionally, one malaria test-positive case in facility A was not referred, given artesunate or ACTs because the provider opted to administer paracetamol whilst waiting other test results. | - *“Yes, because before* [SS] *when faced with a fever we gave ACTs without doing the mRDT, but now the supervisor told us the mRDT should be positive before giving ACTs”.* - *“If you do not refer…you yourself know that if the child dies, it is that you have contributed to their death. SS showed us that handing over* [the case] *to someone is not a weakness, we used to see it as a weakness. That's what used to traumatize us, but since SS we refer* [cases]*. It's a skill to refer in time, but before we didn't know it was a skill.”* - *“Supportive supervision made the difference between simple malaria and severe malaria clear, which I put into practice.”* |
| Diagnosing and treating malaria test-negative cases in children <5 years | - When confronted with malaria test-negative cases, providers typically pursued alternative diagnoses by conducting additional tests to ascertain the cause of the fever. The most common diagnosis among these cases were respiratory tract infections marked by the presence of a cough. In these cases, providers mainly administered paracetamol (total: 35/65); antibiotics (total 22/65); mebendazole (10/65); aspirin (total 5/65); vitamins (total 4/65), and/or ORS (total 3/65), - However, one mRDT-negative case in facility C was diagnosed with malnutrition and given Coartem, Amoxicillin, Mebendazole and Plumpy’nut. In this case, the provider did not diagnose the case with malaria and did not request malaria microscopy yet mentioned that their decision was influenced by their SS supervisor’s advice to explore explanations other than malaria for fevers in mRDT negative cases. - Also, four mRDT negative cases were diagnosed with uncomplicated malaria whilst providers awaited malaria microscopy results that were postponed due to a temporary unavailability of laboratory services. Additionally, in facility D, an mRDT-positive case with a negative malaria microscopy was diagnosed as malaria solely based on the positive mRDT. Consequently, an ACT was administered. | - *“The advice received from the supervisors influenced* [my] *decision by taking care to explore other illnesses… The supervisors recommended that I take an integrated approach to case management.”* - *“With the negative mRDT, you really need to push the clinical investigations further, which is where the advice from supportive supervision influenced my decision.”* - *“The supportive supervision led us to give advice to the mother. That's why I advised the mom to avoid self-medication.”* |
